# Supplementary material for: Perception of the Role of Food and Dietary Modifications in Patients with Inflammatory Bowel Disease: Impact on Lifestyle
Source: Nutrients. 2021 Feb 26;13(3):759. doi: 10.3390/nu13030759 (PMC7996868; doi:10.3390/nu13030759)
Supplement: Supplementary file 1 [file nutrients-13-00759-s001.pdf]

Date____________________________

Section A) Personal data and basic information

A.1) Surname ______________________________

A.2) First name _________________________________

A.3) Date of birth __________________________

A.4) Telephone number _______________________________

A.5) Age ◻<18 ◻ 18-39 ◻ 40-65 ◻ >65

A.6) Gender ◻ Male ◻ Female

A.7) Weight _______________

A.8) Height _____________

A.9) Ethnicity ◻ Caucasian ◻ African ◻ Asian

A.10) Religion ◻ Christian religion ◻ Muslim religion

◻ Other (specify)________________________

A.11) City ________________________________

A.12) Marital status ◻ Single ◻ Married

A.13) Smoker ◻ Yes ◻ No ◻ Ex-smoker

A.14) Qualification ◻ No qualification ◻ Primary School Degree ◻ Secondary School Degree ◻ High school Degree ◻ University Degree

A.15) Employment

◻ Student

◻ Employee

◻ Freelance/entrepreneur

◻ Workman, artisan

◻ Unemployed

◻ Jobseeker

◻ Unfit for work

◻ Housewife/household

◻ Retired

◻ Other (specify) _____________________

Section B) Characteristics of the disease

B.1) Diagnosis ◻ Ulcerative colitis ◻ Crohn's disease

B.2) Date of diagnosis ______________________

B.3) Duration of the disease (years) ◻<5 ◻ 5-10 ◻ 11-20 ◻ >20

B.4) Disease activity (at the time of the interview) ◻ Remission ◻ Mild activity ◻ Moderate ◻ Severe

B.5) Do you take any medication? □ Yes □ No
And, if yes, which one?

____

____

____

B.6) Have you had surgery due to the disease? □ Yes □ No

And, if yes, which one?

____

Section C) Dietary habits

C.1) Do you think that nutrition could be a cause of the disease? □ Yes □ No

C.2) Have you changed your diet since the diagnosis? □ Yes □ No

C.3) Have you noticed any changes in symptoms since your dietary modification? □ Yes □No

C.4) Do you follow a specific diet? □ Yes □ No

And, if yes, which one?

____

C.5) Are you satisfied with your diet? □ Yes □ No

C.6) Do you take any food supplements? □ Yes □ No

And, if yes, which ones?

____

____

C.7) Are you satisfied with your weight? □ Yes □ No

C.8) Have you had the same weight since diagnosis?

- □ Yes
- □ No, I lost weight
- □ No, I have been gaining weight

C.9) Did you find any foods or drinks that trigger the symptoms? □ Yes □ No
C.10) Did you avoid foods since diagnosis? □ Yes □ No
Do you always avoid them or only during relapses? □ Always □ Only during relapses

C.11) Which foods do you prefer to eat and which avoid?

	I don't like	I avoid it because it triggers the symptoms	I eat it despite the symptoms	I avoid it because suggested by the doctor	I eat it without difficulty
Spicy food	□	□	□	□	□
Seasoned food	□	□	□	□	□
Fried food	□	□	□	□	□
Carbonated drinks	□	□	□	□	□
Milk and dairy products	□	□	□	□	□
Energy drink	□	□	□	□	□
Alcoholic drink	□	□	□	□	□
Pork	□	□	□	□	□
Processed meat	□	□	□	□	□
Vegetable	□	□	□	□	□
Fruit	□	□	□	□	□
Legumes	□	□	□	□	□
Whole grain bread	□	□	□	□	□
Bread	□	□	□	□	□
Eggs	□	□	□	□	□
Rice	□	□	□	□	□
Chicken	□	□	□	□	□
Pasta	□	□	□	□	□
Fish	□	□	□	□	□
Coffee	□	□	□	□	□
Candy	□	□	□	□	□

C.12) Has the disease modified your daily activities? □ Yes □ No

C.13) Has the disease changed your social life? (for example eating out with family or friends?)
□ Yes □ No

C.14) Did you change your lifestyle due to the disease? □ Yes □ No

And if yes, how?

□ Small and frequent meals
□ I started doing sport
□ I avoid sport
□ Relaxation techniques (for example meditation)
□ Other____

C.15) In your opinion, compared to drugs nutrition is:

□ more important □ equally important □ less important □ it is not important

C.16) Has your drugs intake affected your appetite?

□ I am hungrier □ I am less hungry □ No change

C.17) In case of surgery, have you modified your diet? □ Yes □ No
And if yes, how?____

UNIT D) Food intolerance

D.1) Are you on a gluten free diet? □ Yes □ No
D.2) Have you ever performed tests for gluten intolerance? □ Yes □ No
D.3) And if yes, which? □ Antibodies
□ Biopsy
□ Other (specify) ___________________

D.4) And if not, why are you on a gluten free diet?
- □ Gluten cause symptoms
- □ Suggested by the doctor
- □ Advice from friends or relatives
- □ Other________________________________

D.5) Which symptoms do you usually experience taking gluten?
- □ No symptoms
- □ Bloating
- □ Abdominal pain
- □ Loss of appetite
- □ Diarrhea
- □ Constipation
- □ Belching
- □ Flatulence
- □ Asthenia
- □ Nausea and/or vomiting
- □ Other______________________________

D.6) Are you on a lactose free diet? □ Yes □ No
D.7) Have you ever performed tests for lactose intolerance? □ Yes □ No
D.8) And if yes, which?
- □ Breath test
- □ Other___________________________

D.9) And if not, why are you on a lactose free diet?
- □ Foods with lactose causing symptoms
- □ Suggested by the doctor
- □ Advice from friends or relatives
- □ Other________________________________

D.10) Which symptons do you usually experience taking lactose-containing foods?
- □ No symptoms
- □ Bloating
- □ Abdominal pain
- □ Loss of appetite
- □ Diarrhea
- □ Constipation
- □ Belching
- □ Flatulence
- □ Asthenia
- □ Nausea and/or vomiting
- □ Other______________________________
